# Successful treatment of highly recurrent facial baroparesis in a frequent high-altitude traveler: a case report

**DOI:** 10.1186/s13256-020-02557-9

**Published:** 2020-11-12

**Authors:** Jason P. Caffrey, Jason W. Adams, Isabel Costantino, Kristin Klepper, Elina Kari, Lori A. Brown

**Affiliations:** 1 School of Medicine, University of California, San Diego, 9500 Gilman Drive, La Jolla, CA 92093 USA; 2 Department of Neurosciences, School of Medicine, University of California, San Diego, 9500 Gilman Drive, La Jolla, CA 92093 USA; 3 Center for Academic Research and Training in Anthropogeny, University of California, San Diego, 9500 Gilman Drive, La Jolla, CA 92093 USA; 4 Division of Otolaryngology, Head and Neck Surgery, Department of Surgery, University of California, San Diego, 9444 Medical Center Drive, La Jolla, CA 92037 USA; 5 Division of General Internal Medicine, Department of Medicine, University of California, San Diego, 200 West Arbor Drive, San Diego, CA 92103-8415 USA

**Keywords:** Altitude and aviation medicine, Baroparesis, Cranial nerve trauma, Facial nerve palsy, Facial paralysis

## Abstract

**Background:**

Facial baroparesis is a palsy of the seventh cranial nerve resulting from increased pressure compressing the nerve along its course through the middle ear cavity. It is a rare condition, most commonly reported in barotraumatic environments, in particular scuba diving and high-altitude air travel. We report here an unusual case of highly frequent baroparesis, workup, and successful treatment.

**Case presentation:**

A 57-year-old Caucasian male frequent commercial airline traveler presented with a 4-year history of recurrent episodes of right-sided facial paralysis and otalgia, increasing in both frequency and severity. Incidents occurred almost exclusively during rapid altitude changes in aircraft, mostly ascent, but also during rapid altitude change in an automobile. Self-treatment included nasal and oral decongestants, nasal corticosteroids, and warm packs. Temporal bone computed tomography (CT) scan revealed possible right-sided dehiscence of the tympanic bone segment; audiogram and magnetic resonance imaging of the internal auditory canals were unremarkable. After a diagnosis of facial nerve baroparesis was made, the patient underwent myringotomy with insertion of a pressure equalization tube (PET) into the right tympanic membrane. Despite re-exposure to altitude change multiple times weekly post-treatment, the patient reported being symptom-free for more than 6 months following intervention.

**Conclusions:**

Prompt PET insertion may represent the preferred treatment for individuals who suffer recurrent episodes of facial baroparesis. Education regarding this rare condition may prevent unnecessary testing and treatment of affected patients. Future studies should explore the pathophysiology and risk factors, compare therapeutic options, and provide follow-up data to optimize the management of affected patients.

## Background

Facial baroparesis is a palsy of the seventh cranial nerve (CN) resulting from increased pressure compressing the nerve along its course through the middle ear cavity. Cases of facial baroparesis have been sparsely reported, most commonly in settings of increased barotrauma, such as scuba diving [1–3] and high-altitude air travel [4–11]. A rare phenomenon, facial baroparesis is thought to result from ischemic neuropraxia of CN VII as it passes through the tympanic segment of the facial (Fallopian) canal. Impaired baroregulation of the Eustachian tube coincides with a defect in the wall of the facial nerve canal, likely resulting in ischemic compression of CN VII in the middle ear cavity. Facial canal dehiscence is a variant anatomic finding: In one anatomic series, facial nerve exposure was found in 12–26% of temporal bones [12, 13]. Although dehiscence of CN VII in the facial canal is well described, high-resolution CT scanning is imperfect at detecting this finding [14]. Limited animal model studies have demonstrated compression-induced transient neuropraxia of the facial nerve [15].

A literature search yielded ten published case reports of facial baroparesis associated with high altitude (excluding patients with a history of middle ear structural abnormalities or postoperative complications), most of which were either acutely self-resolving or treated medically. Only four cases used interventional treatment (ventilation tube placement) to prevent recurrence, and these patients had only six or fewer previous episodes. We report here a case of high-frequency facial baroparesis during rapid altitude change in both commercial flights and high-altitude mountain driving that resolved with myringotomy and placement of a pressure equalization tube (PET).

## Case presentation

A 57-year-old Caucasian man presented to his primary care physician with a 4-year history of recurrent, transient right-sided facial paralysis and otalgia in settings of rapid altitude change. Episodes began with an inability to equalize ear pressure in his right ear shortly after commercial aircraft take-off followed by right-sided otalgia, unilateral right-sided facial droop, paralysis, and numbness, usually before reaching cruising altitude. He denied hearing loss between episodes, tinnitus, vertigo, left-sided ear symptoms, or symptoms at any times other than changing altitude. Notably, the patient traveled up to nine times weekly aboard domestic commercial flights. His episodes occurred with the highest frequency during aircraft ascent, but one incident occurred during aircraft descent and another during automobile travel from an altitude of 5000 to 11,000 feet over 90 minutes. Likelihood of paralysis increased when his trip coincided with periods of nasal congestion due to viral upper respiratory infection or seasonal allergies, but it did not correlate with the number of minutes at high altitude, type of aircraft, or frequency of travel per week. At the time of initial clinic presentation, he described escalating frequency and severity of baroparetic episodes but denied any prior medical evaluation.

Initial self-treatment during flights included yawning and performing the Toynbee maneuver (swallowing with a pinched nose) to equalize ear pressures, applying pressure with a hot water bottle, and the usage of over-the-counter (OTC) nasal decongestant sprays, which relieved pain and paralysis within minutes. He eventually began to pre-medicate with decongestants before flights with variable success.

The physical and neurological examinations on the patient’s first visit were as follows. Physical examination revealed a middle-aged man in no apparent distress. Blood pressure was 118 systolic/70 diastolic, heart rate 68, respiratory rate 14, temperature 98.0°F, and oxygen saturation 98% on room air. The head was normocephalic and atraumatic, the pupils were equally reactive and responsive to light, and extraocular movements were intact. Sinuses were nontender, nares were patent with septal deviation present, and nasal mucosae were normal. Examination of the ears revealed normal canals and tympanic membranes without tympanosclerosis, retraction, or hemotympanum. Hearing was normal bilaterally, and results on the Rinne and Weber tests were unremarkable. Cranial nerves II–XII were intact, including comprehensive facial nerve testing with normal findings, and the remainder of the neurological examination was likewise unremarkable.

Past medical history was notable for mild intermittent allergic rhinitis, well-controlled hypothyroidism, obesity (body mass index 38), obstructive sleep apnea well controlled on continuous positive airway pressure, and dyslipidemia. Past medical history was negative for prior chronic otitis media or any previous ear, sinus, or nasal surgeries. Medications prior to the present diagnosis included levothyroxine, vitamin D supplementation, a testosterone patch, and as-needed OTC nasal and oral decongestants. Between episodes, detailed head, neck, and neurological examinations were within normal limits and without evidence of any residual facial weakness or evidence of chronic or prior otitis media.

The patient was raised and currently resides in California and is a married self-employed attorney with two children. The patient’s parents and two siblings are alive and reportedly healthy. He denied any family history of cancers, cardiovascular, or neurological diseases and likewise reported experiencing no unusual environmental exposures. The patient reported rare, light alcohol intake but was a lifetime non-smoker and did not use any recreational drugs.

**Fig. 1:**
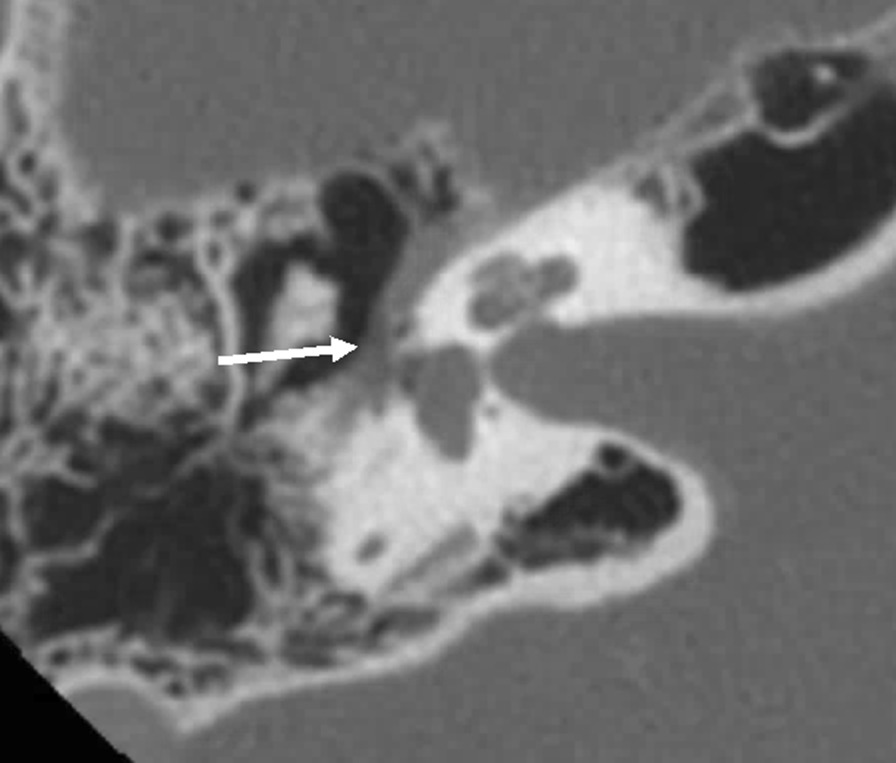
Right-sided temporal bone CT scan. Axial presentation shows dehiscence of the tympanic portion of the right facial nerve (white arrow)

Laboratory tests, including thyroid stimulating hormone, were unremarkable. Audiogram demonstrated no evidence of sensorineural hearing loss and normal flexibility and mobility of the tympanic membrane. Magnetic resonance imaging of the internal auditory canals with and without contrast revealed no masses or other lesions in the internal auditory canal or at the cerebellopontine angle and were without suspicious enhancement of the facial nerve. CT of the temporal bones suggested right-sided dehiscence of the facial canal and showed no evidence of sinus abnormalities or chronic infection (Fig. 1).

Pending imaging and specialist evaluation, nasal corticosteroids were added to his regimen. The patient was referred to otolaryngology and underwent myringotomy with insertion of a titanium PET into the right tympanic membrane. At 2-month follow-up after ear tube placement, the patient was asymptomatic and the PET was found to be stable and patent. At 6-month follow-up, the patient reported having flown multiple times weekly without needing to pre-medicate and without recurrence of symptoms, and the PET remained stable and patent.

## Discussion

We present here a case of highly recurrent facial baroparesis in a frequent high-altitude traveler who was successfully treated by PET insertion into the right tympanic membrane. A 57-year-old Caucasian man without uncommon past medical history presented with a 4-year history of recurrent, transient, right-sided facial paralysis and otalgia in settings of rapid altitude change, most commonly during ascent in commercial aircraft. Shortly after take-off, the patient experienced an inability to equalize ear pressure in his right ear, followed by right-sided otalgia, unilateral right-sided facial droop, paralysis, and numbness. With mild success, the patient initially attempted self-treatment during flights, which included yawning and the Toynbee maneuver to equalize ear pressures, applying pressure with a hot water bottle, and OTC nasal decongestants. A temporal bone CT scan indicated right-sided dehiscence of the  facial canal, and the patient underwent myringotomy with insertion of a titanium PET into the right tympanic membrane. At 2- and 6-month follow-up visits with his primary care physician, the patient reported having flown multiple times weekly, yet he was asymptomatic and the PET was stable and patent. Thus, PET insertion may provide a valuable therapeutic option for patients who experience recurrent facial baroparesis but are unable to alter the frequencies of their exposures to rapid altitude changes.

This patient exhibits many unique characteristics of facial baroparesis compared to the existing literature. He experienced highly frequent, recurrent episodes over a multi-year period. Previous case reports, in contrast, describe isolated single or limited repeated incidents. Whereas most previous cases were treated medically or were self-limited and untreated, our patient underwent myringotomy and PET placement. Other reports of PET placement were in patients with few occurrences and limited flight frequency and included only limited follow-up data. With our case, in contrast, flight frequency remained high, and follow-up data confirm treatment efficacy more than 6 months post-intervention.

A few aspects of our patient's presentation are consistent with the limited number of past case reports. As with others, our patient primarily experienced symptoms during altitude changes, experienced inability to equalize his ear pressure, and experienced otalgia preceding transient unilateral facial weakness. Similarly, two prior case reports describe symptoms during high-altitude automobile travel. However, one of these cases had other clinical findings—including other CT abnormalities and prolonged hearing loss—suggesting the presence of another causal or comorbid pathology [6, 9]. Other case reports have also pointed to dehiscence of the facial canal on CT [4, 11], but these cases also describe additional symptoms including vertigo, hyperacusis, and tinnitus, unlike our patient.

The causes and risk factors associated with facial baroparesis are not well understood and present an opportunity for further investigation. Dehiscence of the facial nerve in the facial canal is not a rare finding, although most case reports of baroparesis describe a single occurrence of symptoms rather than recurrence, as was experienced by our patient. Other factors have been hypothesized to contribute to the risk of nerve injury in barotraumatic environments, including upper respiratory infections or even neurotropic viruses [5]. The long-term potential for facial nerve damage due to untreated, highly recurrent episodes of baroparesis is unknown. With the limited number of cases reported to date, rigorous comparison of demographics, risk factors, patient history, and efficacy of treatment approaches is difficult. There is no evidence-based optimal treatment of patients with this condition. PET placement may not represent a valid plan of care for all patients, and the long-term follow-up of patients with this condition is not described in the literature.

Education about this rare condition may prevent unnecessary and costly emergency workup for affected patients. For example, when not recognized in a diver, the condition can be mistaken for an air embolism, resulting in inappropriate recompression treatment, restricted diving, and other testing and treatment. Another reported case resulted in the emergency landing of a commercial aircraft followed by a full emergency hospital stroke evaluation including extensive neuroimaging and an overnight stay [16]. Clinical cases of facial baroparesis may appear infrequently, but increased awareness about this condition is clearly warranted regardless. In addition to increasing awareness, future studies should explore the pathophysiology and risk factors, compare therapeutic options, and longitudinally follow patients to further enhance the understanding and management of this rare condition.

## Conclusions

In a case of highly recurrent, chronic facial baroparesis during airline travel and high-altitude automobile driving, prompt treatment with PET insertion offered immediate and complete resolution of symptoms for at least 6 months despite frequent recurrent exposure to rapid altitude change. The finding of facial canal dehiscence on high-resolution CT scan may be an underlying anatomic variant associated with risk for this rare condition. Unless contraindicated on a case-specific basis, PET insertion represents a preferred treatment for patients with recurrent episodes of facial baroparesis.

## Data Availability

Electronic Health Record at UCSD Health.

## Abbreviations

CN: Cranial nerve
CT: Computed tomography
OTC: Over-the-counter
PET: Pressure equalization tube
